# Gastrointestinal nematodes of European wild boar from distinct agricultural and forest habitats in Poland

**DOI:** 10.1186/s13028-020-0508-7

**Published:** 2020-02-05

**Authors:** Paweł Nosal, Jerzy Kowal, Anna Wyrobisz-Papiewska, Marek Wajdzik

**Affiliations:** 1 Department of Environmental Zoology, Institute of Animal Sciences, Faculty of Animal Sciences, University of Agriculture in Krakow, Mickiewicza av. 24/28, 30-059 Kraków, Poland; 2 Department of Natural and Cultural Heritage, Animal Ecology and Wildlife Management, Faculty of Forestry, University of Agriculture in Krakow, 29 Listopada av. 46, 31-425 Kraków, Poland

**Keywords:** Arable lands, Forests, Helminths, *Sus scrofa*

## Abstract

The study aimed to compare the gastrointestinal helminthofauna of free-ranging wild boars from arable lands and forests, which are the natural habitats for wild boar in Poland and further to investigate if wild boars living in agricultural environments could acquire helminths commonly detected in domestic pigs. In 2011–2014, a total of 57 wild boars were examined post-mortem for the presence of gastrointestinal nematodes. Altogether, all but two of the animals were infected, and seven nematode species were found. The mean infection burden was 68.9 parasites, ranging from 1 to 381 worms. In forest areas, *Ascarops strongylina*, *Physocephalus sexalatus*, and *Globocephalus urosubulatus* were common, whereas on arable lands, the animals were more frequently infected (P < 0.05) by *Ascaris suum* and *Trichuris suis*, which are parasites that commonly occur in domestic pigs. *Oesophagostomum dentatum* was observed only in wild boars on arable lands, and *Bourgelatia diducta*, which is alien to European suids, appeared irrespective of habitat type. These results show significant differences in parasite spectra among wild boars living in forests or arable lands in Poland and indicates the risks of parasite transfer from domestic pigs to free-ranging wild boars. Furthermore, in farmed game, organic farming, or in the case of agritourism farms, one should be aware of the risk of related animals acquiring new and alien parasite infections by being kept outdoors.

## Findings

Since the 1990s the wild boar (*Sus scrofa scrofa*) population has increased remarkably in most areas of Europe, including Poland [1, 2]. At present, wild boars move from their primary forest habitat to settling into the agricultural landscape. This results in an increased risk of infectious diseases spreading from wild boars to domestic pigs (*Sus scrofa domestica*) and vice versa. For gastrointestinal nematodes (GINs), this would be of particularly concern if they are zoonotic. The transmission of GINs between wild and domestic suids may especially be enhanced by the extensive animal husbandry implemented in Poland in the form of organic production and agritourism farms. Therefore, the study aimed to compare the worm burden of free-ranging wild boars originating from the two habitats (agricultural lands and primary forests) to investigate if wild boars living in agricultural environments could become infected with GIN species commonly occurring in domestic pigs.

**Fig. 1:**
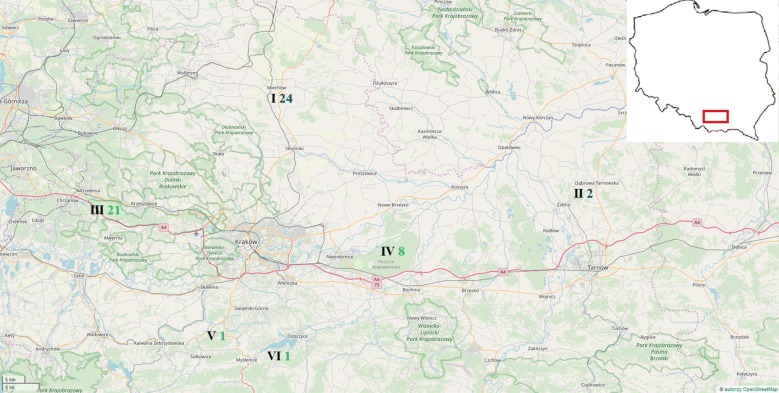
Geographical location of the Polish agricultural (I–II) and forest (III–VI) hunting districts of the harvested wild boars. Hunting grounds: I—Miechów (50° 21′ 23″ N, 20° 01′ 40″ E), II—Dąbrowa Tarnowska (50° 10′ 28″ N, 20° 59′ 10″ E), III—Dulowa Primeval Forest (50° 7′ 27″ N, 19° 31′ 13 E″), IV—Niepołomice Primeval Forest (50° 01′ 44″ N, 20° 20′ 44″ E), V and VI –Myślenice (49° 50′ 01″ N, 19° 56′ 17″ E). Digits represent the number of wild boars collected in arable lands (blue color) or forests (green)

The Polish Hunting Law [3] defines arable lands are areas with less than 40% of forest, whereas forested areas have at least 40% of forest. The wild boars originated from six hunting districts: four representing arable land habitats (average forest cover 1–23.5%; most animals were harvested near Miechów—9.5% of forest cover), and two forested ones, located in the Niepołomice and Dulowa Primeval Forests (average forest cover of 96.6% and 68.7%, respectively) (Fig. 1).

A total of 57 wild boars were shot during three consecutive hunting seasons (from December to January of 2011–2014) and examined post-mortem. The entire digestive tract was removed from the carcass [4], whereafter all isolated nematodes were identified by their morphological features [5–7]. Initially, the alimentary tract was divided into three compartments, i.e. stomach, small, and large intestine; individual segments were then cut longitudinally and the entire content was washed out on a 125 μm sieve. The material gathered on the sieve was flushed with a solution of physiological saline into a 500 mL container. Subsequently, the solution was poured out onto a black tray and the helminths were collected from the suspension. Additionally, the mucus membrane of the stomach and small intestine was scraped (after being rinsed with warm water) using a blunt knife, then flushed onto a sieve and examined under a dissecting microscope. The collected nematodes were preserved in 70% ethanol, transferred onto glycerine-based microscopic slides, and then identified by the dimensions and shape of the body, buccal capsule (for oesophagostomins, the number of leaves in the *corona radiata* was counted), oesophagus and tail, using previously published descriptions [5–7]. Based on tooth eruptions and replacement patterns [8], the wild boars were categorized into age groups: juveniles (< 1 year old) and adults (≥ 1 year old).

Prevalence (P), mean intensity (I), and mean abundance (A) of GIN infections were calculated according to Bush et al. [9]. The Quantitative Parasitology Web [10] was used to compare the prevalence of infection (Pearson’s chi-squared test), or the quantitative I and A variables of infection, in relation to the wild boars’ site of origin, sex, and age group. Furthermore, the similarities between the parasite communities in hosts from different habitats were compared using the Bray–Curtis cluster method to obtain a group average link on the non-standardized, non-transformed data (the intensity of infection used as the input data) in the BioDiversity Professional program [11].

GINs were present in all animals from the forest sites (n = 31), and in 24 out of 26 wild boars from the arable lands (Table 1). Hence, GINs occurred in 96.5% of the wild boars examined. The mean number of GINs per animal was 68.9 (range 1–381), and the animals were infected with one to four species each. Altogether, seven species of GINs were detected. In the stomachs, *Ascarops strongylina* and *Physocephalus sexalatus* of the Spirurida order were found while in the intestines, *Ascaris suum*, *Trichuris suis*, and *Globocephalus urosubulatus*, *Oesophagostomum dentatum* and *Bourgelatia diducta* were detected.

**Table 1:** Gastrointestinal nematode infection (P, %—prevalence, I—mean intensity, R—range, A—mean abundance) in wild boars by hunting area

Hunting area and number of wild boars (n)	*Ascarops strongylina*	*Physocephalus sexalatus*	*Globocephalus urosubulatus*	*Ascarissuum*	*Trichurissuis*	*Oesophagostomum dentatum*	*Bourgelatia diducta*
Forest sites (n = 31^a^)							
P (%)	82.6^b^	87.0	93.5^b^	6.5^b^	48.4^b^	0.0	19.4
I	26.5	32.5	79.1	1.0	8.1	0.0	4.8
R	1–71	2–191	2–312	1	1–53^b^	0	1–11
A	21.9^b^	28.2	74.0^b^	0.1^b^	3.9	0.0	0.9
Arable lands (n = 26)							
P (%)	3.8^c^	0.0	7.7 ^c^	61.5^c^	84.6^c^	3.8	23.1
I	2.0	0.0	13.0	1.6	4.2	26.0	3.2
R	2	0	11–15	1–5	1-23^c^	26	1–6
A	0.1^c^	0.0	1.0^c^	1.0^c^	3.5	1.0	0.7

^a^Eight animals from forest sites, i.e. a female < 1 year, two males < 1 year, and five males ≥ 1 year old were obtained without stomachs, which was taken into account when estimating the infection of wild boars with *A. strongylina* and *P. sexalatus*—the gastric parasite species

^b, c^In same column, different superscript letters between particular infection rates (P, R, or A) mean significant difference at P < 0.05

The comparison of parasitological data from the different habitats are shown in Table 1. Apart from the absence of *O. dentatum* in the wild boars from forest areas, the GINs which significantly prevailed there, were the Ascaropsinae and the blood-sucking *Globocephalus* (P < 0.05). In contrast, in the arable lands, the wild boars were significantly (P < 0.05) much more infected by GINs that commonly occur in domestic pigs, i.e. *A. suum* and *T. suis*. *Bourgelatia diducta* was observed in wild boars irrespective of the habitat.

**Fig. 2:**
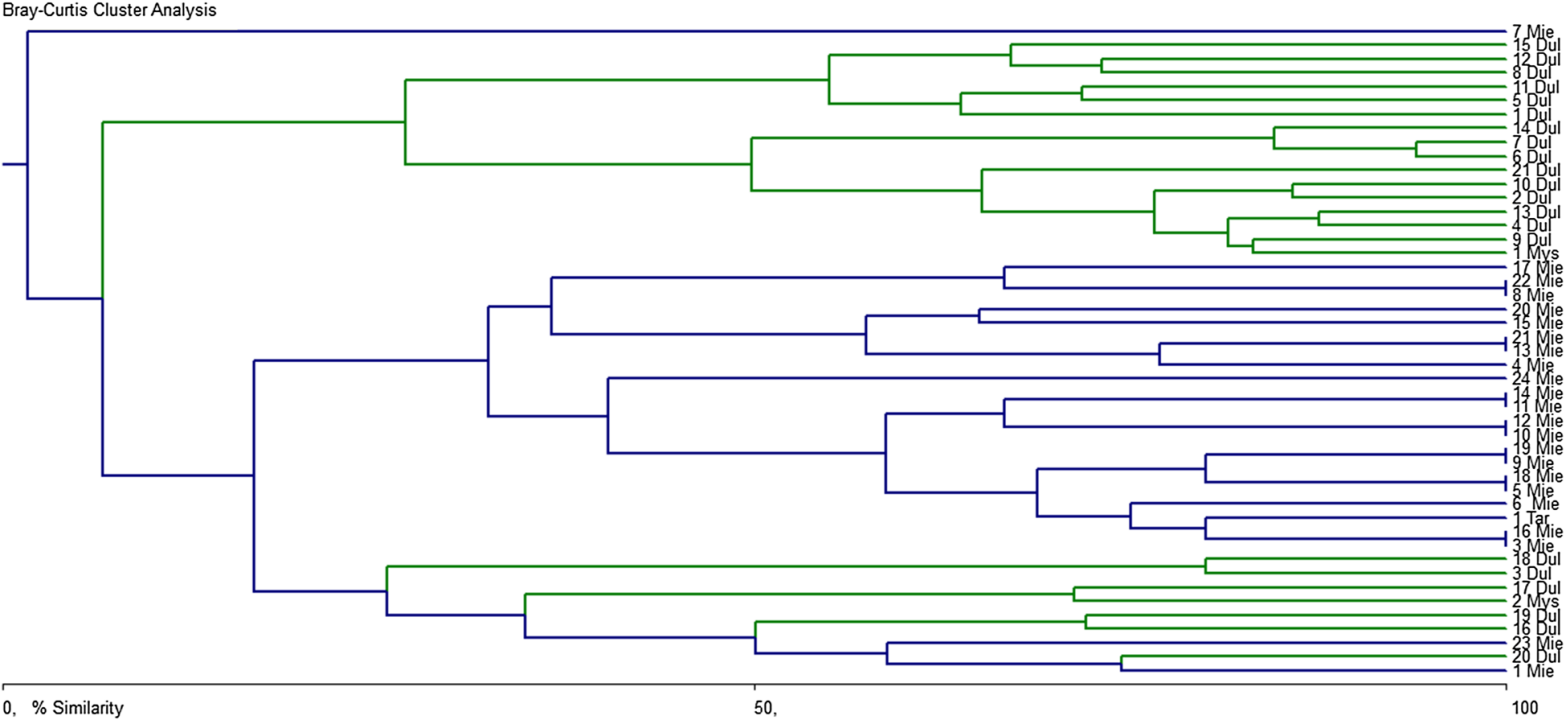
Cluster analysis dendrogram of gastrointestinal nematode species composition in wild boars from different habitats, based on Bray–Curtis similarity index (a group average link, the intensity of infection as the input data). “Dul”, “Mie”, “Mys”, “Tar” placed behind the numbers stand for animals originating from the hunting districts in Dulowa, Miechów, Myślenice, and Dąbrowa Tarnowska, respectively. Animals (n = 47; two uninfected, eight from Niepołomice without stomach) from the arable habitats are shown in blue color; those from forest areas are indicated by green color

The differences between the habitats are illustrated in Fig. 2. The cluster analysis confirmed the qualitative and quantitative differences of the GINs community structure in the wild boars representing the two considered habitat types. The core of the community was clearly divided into two groups (6% of similarity): the first formed by wild boars originating from only the forest habitat; and the second which was divided (16.75% of similarity) into two subgroups: one containing wild boars from the forest, and one of wild boars mostly from arable land.

As regards the age group and sex of the animals, there were no statistical differences in GIN infections, although males (n = 29, including 18 adult ones) seemed to have higher parasitic burden than females (n = 28, including 13 adults), and adults (n = 31) harboured more GIN specimens than juveniles (n = 26), apart from the roundworm *A. suum* and whipworm *T. suis*—a finding probably linked to their immunogenicity [12].

In Poland, suids are generally the hosts of nine GIN species: *A. strongylina*, *P. sexalatus*, *Strongyloides ransomi*,* G. urosubulatus*, *A. suum*, *T. suis*, *O. dentatum*, *Oesophagostomum quadrispinulatum* and *B. diducta* [5, 13–16], although so far *S. ransomi* and *O. quadrispinulatum* have been observed exclusively in domestic pigs, and *A. strongylina*, *G. urosubulatus* and *B. diducta* solely in wild boars. The nematode *Hyostrongylus rubidus*, occurring in neighboring Germany [17] and other European countries [18], has never been reported in Poland.

In the present study, the higher prevalence of wild boar in agricultural habitats being infected with *A. suum* and *T. suis* was probably caused by contamination of arable lands with the eggs of these parasites. The eggs of these species are often found in organic fertilizers, and are characterized by a very high viability and resistance to environmental factors [19]. The eggs can also be spread along with surface waters [20], which should cause concern, especially due to the zoonotic potential of roundworms [21]. On the other hand, the eggs and larval stages of *Oesophagostomum* spp. are less resistant to environmental breakdown [22]. Environmental inactivation may therefore explain the observed low prevalence of *Oesophagostomum* spp. infection in wild boars as *Oesophagostomum* spp. are also common in domestic pigs.

Unlike in the wild boars from arable lands, *G. urosubulatus*, *A. strongylida* and *P. sexalatus* dominated the GIN fauna of wild boars from the forests. Forests have better conditions for coprophagic beetles, which are the intermediate hosts of the Spirurida, while the insects on arable lands are probably reduced in numbers by the insecticides used to protect plants. The difference in the burden of *G. urosubulatus* infections (Table 1) may result from a higher density of hosts in forests (1.7 individuals per 100 ha, compared with 0.6 on arable lands), or the higher humidity and thermal stability in forest areas, which favor larval survival [23].

The cause of lack of *O. dentatum* infection in the wild boars in forests with concomitant presence of *B. diducta* in wild boars from both types of habitats remains hypothetical, but it may be due to the existence of antagonistic interactions between these related GIN species. It is known from regions where *B. diducta* is endemic that co-infections with other GINs occur [24, 25]. Nevertheless, taking into account the process of the adaptation of a recently introduced nematode to a new host, it can be assumed that *B. diducta* will also affect the native parasite populations of *S. scrofa* [16].

Until now, there has been a lack of comparative studies on the parasitic fauna of wild boars inhabiting adjacent and distinct forest and agricultural habitats. Rather, researchers have focused on the differences between the parasitic fauna of free-ranging and farmed wild boars [14]. Furthermore, the level of wild boar infection differs in various regions due to e.g. latitude, geographical isolation of the population, presence of larger predators influencing population density, or because of age differences of the host [26–28]. The parasitic fauna of wild boars may also change depending on alterations in environmental conditions.

Although there is a strict focus on effective biosecurity systems in the pig industry due to the risk of transmission of African swine fever [29], also parasites may be spread to domestic pigs, e.g. through a wide variety of biological mechanisms (earthworms, insects, rodents) [30], or mechanically.

An important factor favoring spread of GINs among wild boars is the density of the host population. Therefore, wild boar husbandry in enclosures, agritourism farms holding various species of animals and organic pig management are all systems at high risk of having *Bourgelatia* introduced. Introduction of the GINs could be very harmful in managed or farmed game animals or swine production in Poland.

In conclusion, significant differences in the GIN spectra among wild boars living in forest and arable habitats in Poland were observed. The results suggest that transmission of GINs from domestic pigs to wild boars may occur. This is of particular importance for *A. suum* and *T. suis*, which are both zoonotic. Transmission from wild boars to domestic pigs seems not to occur in the study area due to the confinement of pigs in housing.

## Data Availability

The datasets used and analysed during the current study are available from the corresponding author on reasonable request.

## Abbreviations

A: Mean abundance of infection
GIN: Gastrointestinal nematode
I: Mean intensity of infection
P: Prevalence of infection (%)
R: Range of infection

## References

[1] Kamieniarz R, Panek M (2008). Game animals in Poland at the turn of the 20th and 21st century.

[2] Popczyk B, Popczyk B, Kniżewska W (2016). Management of wild boar *Sus scrofa* population in Poland. Zarządzanie populacjami zwierząt.

[3] Act of 13 October 1995 Hunting Law, Journal 1995 No. 147 item 713 https://prawo.sejm.gov.pl/isap.nsf/download.xsp/WDU19951470713/U/D19950713Lj.pdf**(in Polish)**.

[4] Roepstorff A, Nansen P (1998). Epidemiology, diagnosis and control of helminth parasites of swine.

[5] Tarczyński S (1956). Parasitic worms of swine and wild boars in Poland. Acta Parasitol Pol.

[6] Poelvoorde J (1978). Oesophagostomosis in sows. Zbl Vet Med B.

[7] Lichtenfels JR, Anderson RCA, Chabaud G, Willmott S (1980). No. 7. Keys to genera of the superfamily Strongyloidea. CIH keys to the nematode parasites of vertebrates.

[8] Matschke GH (1967). Ageing European wild hogs by dentition. J Wildl Manag.

[9] Bush AO, Lafferty KD, Lotz JM, Shostak AW (1997). Parasitology meets ecology on its own terms: Margolis et al. revisited. J Parasitol.

[10] Reiczigel J, Marozzi M, Fábián I, Rózsa L (2019). Biostatistics for parasitologists—a primer to quantitative parasitology. Trends Parasitol.

[11] McAleece N, Gage JDG, Lambshead PJD, Paterson GLJ. BioDiversity professional statistics analysis software. Oban: The Natural History Museum & The Scottish Association for Marine Science; 1997. https://www.sams.ac.uk/t4-media/sams/pdf/BioDiversity_Pro_notes.pdf.

[12] Murrell KD (1986). Epidemiology, pathogenesis, and control of major swine helminth parasites. Vet Clin North Am Food Anim Pract.

[13] Frączak K (1974). An attempt at determining the role of parasites as a factor controlling the numbers of a wild boar (*Sus scrofa*) population. Wiad Parazytol.

[14] Gadomska K (1981). The qualitative and quantitative structure of the helmithocenosis of wild boar (*Sus scrofa* L.) living in natural and breeding conditions. Acta Parasitol Pol..

[15] Nosal P, Nowosad B, Petryszak A (2007). *Oesophagostomum quadrispinulatum* (Marcone, 1901) Alicata, 1935—a new for Poland parasite of swine. Wiad Parazytol.

[16] Nosal P, Bonczar Z, Kowal J, Nowosad B (2013). Oesophagostominae (Nematoda: Chabertiidae) of suids from southern Poland. Ann Anim Sci.

[17] von Epe C, Spellmeyer O, Stoye M (1997). Untersuchungen zum Endoparasitenbefall bei Wildschweinen aus freier Wildbahn. Z Jagdwissensch.

[18] Gibson D. 2019 Fauna Europaea: Nematoda, Trichostrongylidae. Fauna Europaea version 2019.08. https://fauna-eu.org.

[19] Black MI, Scarpino PV, O'Donnell CJ, Meyer KB, Jones JV, Kaneshiro ES (1982). Survival rates of parasite eggs in sludge during aerobic and anaerobic digestion. Appl Environ Microbiol.

[20] Senqupta ME, Thamsborg SM, Andersen TJ, Olsen A, Dalsgaard A (2011). Sedimentation of helminth eggs in water. Water Res.

[21] Leles D, Gardner SL, Reinhard K, Iniguez A, Araujo A (2012). Are *Ascaris lumbricoides* and *Ascaris suum* a single species?. Parasites Vectors.

[22] Caballero-Hernández AI, Castrejón-Pineda F, Martínez-Gamba R, Angeles-Campos S, Pérez-Rojas M, Buntinx SE (2004). Survival and viability of *Ascaris suum* and *Oesophagostomum dentatum* in ensiled swine faeces. Bioresour Technol.

[23] Stefański W (1963). Veterinary parasitology.

[24] Yadav AK, Tandon V (1989). Nematode parasite infections of domestic pigs in a subtropical and high-rainfall area of India. Vet Parasitol.

[25] Sato H, Suzuki K, Yokoyama M (2008). Visceral helminths of wild boars (*Sus scrofa leucomystax*) in Japan, with special reference to a new species of the genus *Morgascaridia* Inglis, 1958 (Nematoda: Schneidernematidae). J Helminthol.

[26] Humbert JF, Henry C (1989). Studies on the prevalence and the transmission of lung and stomach nematodes of the wild boar (*Sus scrofa*) in France. J Wildl Dis.

[27] De-la Muela N, Hernandez-de-Lujan S, Ferre I (2001). Helminths of wildboar in Spain. J Wildlife Dis.

[28] Järvis T, Kapel CH, Moks E, Talvik H, Mägi E (2007). Helminths of wild boar in the isolated population close to the northern border of its habitat area. Vet Parasitol.

[29] Sur JH (2019). How far can African swine fever spread?. J Vet Sci.

[30] Jacobs DE, Dunn AM, Walker J (1971). Mechanisms for the dispersal of parasitic nematode larvae: rats as potential paratenic hosts for *Oesophagostomum* (Strongyloidea). J Helminthol.

